# Electro‐fermentation triggering population selection in mixed‐culture glycerol fermentation

**DOI:** 10.1111/1751-7915.12747

**Published:** 2017-07-11

**Authors:** Roman Moscoviz, Eric Trably, Nicolas Bernet

**Affiliations:** ^1^ LBE INRA 102 Avenue des étangs 11100 Narbonne France

## Abstract

Electro‐fermentation is a new technique that could be used to influence the global metabolism in mixed‐culture fermentation**.** In this study, a mixed‐culture cathodic electro‐fermentation of glycerol was investigated. Both microbial community structure and metabolic patterns were altered when compared to standard fermentation. This microbial population shift was more significant when the working electrodes were pre‐colonized by *Geobacter sulfurreducens,* before electro‐fermentation. The electro‐fermenting microbial community was more efficient for producing 1,3‐propanediol with an improved yield of 10% when compared with fermentation controls. Such improvement did not require high energy and total electron input represented < 1% of the total electron equivalents provided only by glycerol. A linear model was developed to estimate the individual metabolic pattern of each operational taxonomic unit. Application of this model compared to the experimental results suggests that the changes in global metabolism were supported by bacterial population selection rather than individual metabolism shift. This study shows for the first time that both fermentation pattern and bacterial community composition can be influenced by electro‐fermentation conditions.

## Introduction

Electro‐fermentation (EF) is a recently developed approach that combines fermentation and bio‐electrochemical systems (BESs) to add a supplementary way of control of fermentation patterns (Moscoviz *et al*., 2016a; Schievano *et al*., 2016). It consists in operating a self‐driven fermentation in the presence of polarized electrodes inside the bulk phase. This additional electron sink/source provides many advantages including the possibility to perform unbalanced fermentations [e.g. stoichiometric production of ethanol from glycerol (Flynn *et al*., 2010)] or to affect metabolic regulations directly through intracellular redox pair balance (Moscoviz *et al*., 2016a). Contrary to most other BESs, EF does not necessarily require high energy input and even small current densities can have a great impact on the overall fermentation performances (Choi *et al*., 2014; Kracke *et al*., 2016; Moscoviz et al., 2016a).

In this context, the glycerol fermentation is of special interest because of its high electron content (4.7 moles of electrons per mole of carbon compared to four moles of electrons per mole of carbon for glucose). Glycerol is so reduced that even biomass synthesis generates an excess of intracellular electron carriers (i.e. NADH, NADPH). Most fermentation end‐products (e.g. ethanol, acetate, butyrate and lactate) are also associated with net NADH generation, when produced from glycerol. Indeed, only few pathways having net NADH consumption are available in glycerol fermentation: either H_2_ is produced or glycerol is converted into 1,3‐propanediol (PDO). The latter is a chemical of industrial interest that can be used for the production of resins, cosmetics, solvents and polymers (Zeng and Sabra, 2011), and its production has been the focus of numerous scientific studies (Almeida *et al*., 2012; Lee *et al*., 2015). As supported by a recent in silico study (Kracke and Krömer, 2014), cathodic EF appears to be an appropriate tool to enhance PDO yield because its production is tightly related to intracellular electron balance and redox conditions. Overall, adding a small supplementary electron source (cathodic EF) could provide several benefits such as: (i) a direct dissipation of these extra electrons into PDO (electrosynthesis); (ii) a shift of metabolic patterns towards a more efficient electron dissipation (i.e. PDO production) caused by regulations ensuring NADH/NAD ratio homoeostasis (Choi *et al*., 2014; Kracke *et al*., 2016; Moscoviz et al., 2016a); (iii) a selection of microbial population that is more efficient for electron dissipation and more adapted to reduced conditions; (iv) a disfavoured microbial H_2_ production by electrochemically producing H_2_ at the cathode, therefore enhancing PDO production as sole NADH dissipating pathway.

Until now, only few studies have implemented an EF strategy to enhance PDO production in mixed‐culture glycerol fermentation (Dennis *et al*., 2013; Zhou *et al*., 2013, 2015; Xafenias *et al*., 2015). In a study focusing on glycerol fermentation in batch tests, Zhou et al. (2013) reported that a strongly negative potential of −1.14 V versus Standard Calomel Electrode (SCE) applied at the cathode was able to improve PDO production from 0.25 mol_PDO_  mol^−1^_glycerol_ in conventional fermentation to 0.50 mol_PDO_ mol^−1^_glycerol_ in electro‐fermentation (Zhou *et al*., 2013). In a second study, a cathodic current was applied (chronopotentiometry at −1 and −10 A m^−2^) during continuous glycerol fermentation (Zhou *et al*., 2015). It was found that PDO production positively correlated with the electron input, reaching an average yield of 0.51 ± 0.07 mol_PDO_  mol^−1^_glycerol_ after 3 weeks of operation_._ In addition, Xafenias et al. (Xafenias *et al*., 2015) reached a maximum concentration of 42  g_PDO_ l^−1^ in fed‐batch mode with a production yield of 0.46  mol_PDO_  mol^−1^_glycerol_ while imposing a potential of −1.34 V versus SCE at the cathode. EF performances reported in this study were clearly higher than the fermentation controls, which reached only 18 g l^−1^ of PDO in more than twice the experimental time. However, in these studies, the very low working potential applied led to hydrogen formation from water at the cathode and it was therefore difficult to assess whether the predominant effect of EF was direct or indirect.

The aim of this work was to investigate whether EF could be used as a tool to redirect mixed‐culture fermentation of glycerol using a cathode potential that does not allow abiotic hydrogen production (based on abiotic cyclic voltammetry, see Fig. S7). Impact on both fermentation patterns and bacterial community structure was studied. As a second step, *Geobacter sulfurreducens* pre‐colonized working electrodes were used to evaluate the potential of adding an electro‐active bacterium as biocatalyst between the working electrode and the fermentative bacteria.

## Results

### Impact of electro‐fermentation on metabolic patterns

**Figure 1:**
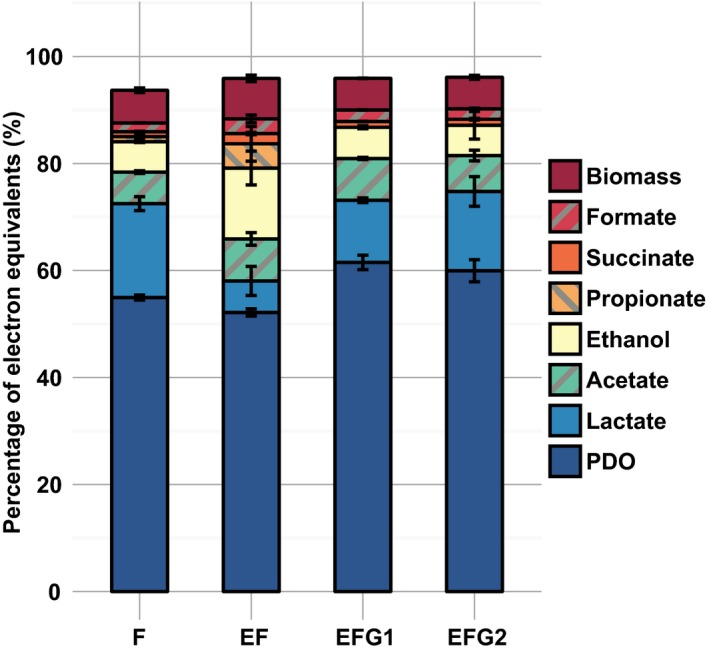
Electron mass balances calculated from the metabolites measured after glycerol depletion in duplicate experiments. Results are normalized on the sum of electron content from initial glycerol and cathodic current. The biomass was estimated from the ATP production associated to the different metabolites production. Error bars represent the minimum and maximum values of the replicates. Calculation is detailed in Appendix S1. F: Classic fermentation. EF: Electro‐fermentation. EFG1‐2: Successive batches of electro‐fermentation with *G. sulfurreducens* pre‐colonized cathode.

Conventional fermentation (F) and electro‐fermentation (EF) were compared in batch pH‐controlled reactors initially fed with 17.5 g_glycerol_ l^−1^. After a lag‐time of about 35 h, probably related to the inoculum storage, both F and EF started and glycerol was nearly depleted within 48 h (83 h since the beginning of experiments, see Figs S2–S3). Electron mass balances closed between 94 and 97% as shown in Fig. 1. The missing part probably corresponds to measurement errors and unmeasured products such as hydrogen. During EF, the cathodic current was always more positive than −0.35 A m^−2^ (see Fig. S6) and the electron input through the cathode represented only 0.2% of the total electron input (i.e. glycerol plus electric current). The electro‐fermentation efficiency η_EF_, an index previously defined by Moscoviz et al. (2016a), was 0.004, meaning that the electric current could not contribute directly for more than 0.4% of PDO production and direct bioelectrosynthesis of PDO was therefore not the predominant reaction (see Appendix S1 for calculations). In both conditions, PDO was the main product and the other by‐products were lactate, acetate and ethanol, being consistent with a previous study carried out with the same inoculum (Moscoviz *et al*., 2016b). Compared to F, EF had nearly no effect towards PDO production since in F and EF, similar yields of 0.48 ± 0.01 mol_PDO_ mol^−1^_glycerol_ and 0.46 ± 0.01 mol_PDO_ mol^−1^_glycerol_ corresponding to final PDO concentrations of 90.7 mM and 86.7 mM were achieved, respectively. Comparable amounts of acetate (5.9 and 7.9% total electron equivalent – TEE – during F and EF, respectively), formate (1.6 and 2.7% TEE, respectively) and succinate (0.9 and 1.9% TEE, respectively) were found in both conditions. However, lactate production in F (17.6% TEE) was higher than its production in EF (5.9% TEE). In contrast, ethanol production was lower in F (5.7% TEE) than EF (13.3% TEE). Traces of propionate and butyrate were only found in EF.

### The addition of *Geobacter sulfurreducens* increased PDO production

Two successive electro‐fermentation series of batch experiment (EFG1 and EFG2) were carried out with *Geobacter sulfurreducens* pre‐colonized WE. The lag phase and fermentation time in EFG1 were about 35 h as observed in F and EF. However, the lag phase in EFG2 was reduced to < 12 h, probably because the biomass inoculated was already active (see Figs S4–S5). Electron mass balances are presented in Fig. 1. and closed at about 96%. Similarly, the cathodic current was always more positive than −0.25 A m^−2^ in EFG1 and was more positive than −0.05 A m^−2^ in EFG2, representing 0.1% and < 0.1% of the total electron input respectively (see Fig. S6). In both conditions, η_EF_ was below 0.001, meaning that the electric current could not contribute directly for more than 0.1% of the PDO production. PDO production increased by about 10% when compared to conventional fermentation and electro‐fermentation without *G. sulfurreducens*. PDO yield in EFG1 and EFG2 were 0.54 ± 0.02 and 0.53 ± 0.02 mol_PDO_ mol^−1^_glycerol_ (61.5 and 60.0% TEE, respectively) respectively. The final PDO concentrations were 102.5 mM and 101.2 mM for EFG1 and EFG2 respectively. In the two batch series, lactate was the main by‐product (11.6–14.8% TEE) along with acetate (6.7–7.8% TEE) and ethanol (5.7–5.8% TEE).

### Fermentative microbial communities in glycerol EF

Microbial community compositions of the inoculum and bulk phase of all experimental conditions were measured using MiSeq sequencing. Relative abundances and affiliations of each operational taxonomic unit (OTU) are provided in Table 1. The inoculum was mainly composed of bacteria from the *Firmicutes* (72.6%), *Bacteroidetes* (20.0%) and *Proteobacteria* (7.2%) phyla and was dominated by OTUs 5 and 1 with 30.5 and 13.4% of the total bacterial community respectively. These two OTUs were related to *Clostridium intestinale* (98% 16S rRNA sequence similarity with OTU5) and *Enterococcus avium* (100% 16S rRNA sequence similarity with OTU1). After substrate depletion, only 15 OTUs had a relative abundance of more than 1% in at least one reactor. OTU1, which was by far the most dominant species within the *Firmicutes* phylum, was found to dominate the bacterial community of EFG1 and EFG2 (47.8 and 54.7% of the total bacterial community respectively). OTU1 also represented a large part of the bacterial community of F and EF (20.1 and 22.7% respectively). OTU1 bulk abundancy in all reactors was correlated with high PDO production yield (*R*² = 0.65, *P* < 0.05, see Fig. S8). OTUs from the *Proteobacteria* phylum were enriched in all the conditions, especially during EF and EFG1 with 19.1 and 25.2% of the final bacterial community, respectively, while they represented only 7.2% of the inoculum. The dominant OTU of the *Proteobacteria* phylum was OTU2, which had 99% 16S rRNA sequence similarity with *Citrobacter freundii*. OTU13 was the second dominant OTU of this phylum and was only found during EFG1 and EFG2 (2.9 and 0.5% of the total bacterial community respectively). OTU13 bulk abundancy positively correlated with PDO production yield (*R*² = 0.53, *P* < 0.01, see Fig. S8). This OTU had 100% of 16s rRNA sequence similarity with species from *Escherichia‐Shigella* genera such as *Shigella sonnei and Escherichia fergusonii*. Species from the *Bacteroidetes* phylum were highly enriched in all conditions (54.8, 38.4 and 32.0% of the total bacterial community for F, EF and EFG2 respectively) except in EFG1 where they represented only 14.7% of the total bacterial community. OTU3 and OTU6 accounted for more than 90% of the *Bacteroidetes* species in all conditions. Nucleotide sequence analyses of the OTU3 16S rRNA genes revealed 98% of sequence homology with *Dysgonomonas mossii* whereas OTU6 was not closely related to any cultured species.

**Table 1:** Clone abundances and identification obtained after sequencing

OTU n°^a^	Putative identification (% 16S rRNA sequence similarity)	Average abundance in the bulk (%)				
		Inoculum	F^c^	EF^c^	EFG1^c^	EFG2^c^
*Firmicutes*						
1	*Enterococcus avium* (100)	13.4 ± 2.5	20.1 ± 4.3	22.7 ± 11.6	47.8 ± 1.2	54.7 ± 2.2
5	*Clostridium intestinale* (98)	30.5 ± 3.1	1.0 ± 0.3	4.0 ± 1.2	1.2 ± 0.3	0.3 ± 0.0
9	*Clostridium celerecrescens* (100)	0.4 ± 0.4	2.2 ± 0.5	0.9 ± 0.1	5.0 ± 1.1	1.8 ± 1.1
12	Uncultured *Lachnospiraceae* sp.	5.4 ± 1.7	1.0 ± 0.2	1.7 ± 0.1	1.6 ± 0.5	0.3 ± 0.2
30	*Clostridium oroticum* (98)	0.4 ± 0.2	1.5 ± 1.5	1.3 ± 1.1	0.0	0.1 ± 0.1
31	*Clostridium propionicum* (99)	0.6 ± 0.4	3.0 ± 0.1	0.7 ± 0.2	0.0	0.0
34	Uncultured *Veillonellaceae* sp.	0.0	0.0	7.5 ± 10.6	0.0	0.0
Sum of all *Firmicutes* b		72.6 ± 0.7	31.9 ± 5.9	42.1 ± 1.7	59.5 ± 2.4	58.0 ± 0.8
*Proteobacteria*						
2	*Citrobacter freundii* (99)	2.0 ± 0.7	8.0 ± 0.4	15.1 ± 0.4	19.0 ± 4.2	6.4 ± 4.5
8	*Stenotrophomonas pavanii* (100)	0.4 ± 0.3	1.8 ± 0.5	1.3 ± 1.1	0.8 ± 0.6	1.7 ± 0.0
13	*Escherichia fergusonii* (100)	0.0	0.0	0.0	2.9 ± 2.1	0.5 ± 0.3
55	*Telmatospirillum siberiense* (98)	0.1 ± 0.1	0.6 ± 0.8	1.1 ± 1.6	0.0	0.0
61	*Pseudomonas aeruginosa* (100)	0.6 ± 0.9	0.0	0.8 ± 1.1	0.0	0.0
Sum of all *Proteobacteria* b		7.2 ± 1.3	11.6 ± 0.5	19.1 ± 1.0	25.2 ± 3.4	9.7 ± 5.1
*Bacteroidetes*						
3	*Dysgonomonas mossii* (98)	2.0 ± 0.4	40.5 ± 23.6	29.0 ± 10.8	0.3 ± 0.0	28.0 ± 1.9
6	Uncultured *Bacteroides* sp.	5.4 ± 0.6	12.9 ± 17.0	8.4 ± 10.9	13.0 ± 5.9	3.7 ± 3.5
11	*Bacteroides graminisolvens* (99)	6.3 ± 1.1	0.8 ± 0.1	0.6 ± 0.4	0.8 ± 0.1	0.2 ± 0.1
Sum of all *Bacteroidetes* b		20.0 ± 0.5	54.8 ± 7.0	38.4 ± 0.7	14.7 ± 5.6	32.0 ± 5.5
*Tenericutes*						
33	Uncultured *Mollicutes* sp.	0.0	1.4 ± 1.6	0.2 ± 0.1	0.6 ± 0.3	0.3 ± 0.3
Sum of all *Tenericutes* b		0.0	1.4 ± 1.6	0.2 ± 0.1	0.6 ± 0.3	0.3 ± 0.3

a Only the clones with a minimum of 1% abundancy in the bulk in at least one condition are reported.

b Including OTUs with < 1% abundancy.

c F: Classic fermentation (open circuit). EF: Electro‐fermentation (applied potential of −900 mV versus SCE). EFG1‐2: Successive batches of electro‐fermentation (applied potential of −900 mV versus SCE) with *G. sulfurreducens* pre‐colonized cathode.

### Estimated metabolic patterns and clustering of the OTUs

**Figure 2:**
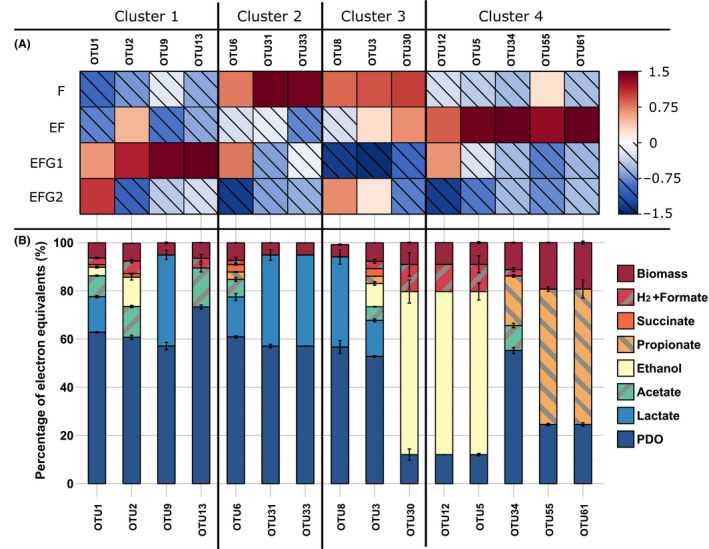
A. Normalized abundancy of all OTUs representing more than 1% of the total bacterial community in at least one reactor (based bulk abundances only). The 0 value corresponds to the experimental mean considering all reactors, the hatched squares correspond to negative values of normalized abundancy (i.e. inferior to the experimental mean), and the plain squares correspond to positive values of normalized abundancy (i.e. superior to the experimental mean). The clusters are the result of k‐mean clustering for *k* = 4 groups. B. Electron mass balances estimated by the model and normalized on the electron equivalent of the glycerol consumed by each OTU. The error bars correspond to the standard deviation of the predicted values obtained by cross‐validation.

As only 15 OTUs accounted for more than 94% of the total bacterial population of the bulk in all conditions, a linear system was written in order to estimate the metabolic profile of each of these OTUs. In order to simplify the analysis of the estimated metabolic patterns, the OTUs were clustered according to their normalized abundancy in bulks using k‐means clustering (*k* = 4) (Fig. 2A). This clustering shows OTUs that were preferentially selected in each condition and must not be confused with raw abundancy heatmap. The metabolic profiles estimated by the model are displayed in Fig. 2B. Cluster 1 was composed of OTUs 1, 2, 9 and 13, which were over‐represented in the bacterial population of EFG1 when compared to the mean abundancy in all conditions. They were all estimated to have a good PDO production yield (> 0.50 mol mol^−1^), especially OTU13, OTU1 and OTU2 that were within the best PDO producers predicted by the model (0.64, 0.55 and 0.53 mol mol^−1^ respectively, see Table S3). The high PDO yield of these OTUs was partly related to their good acetate production, as the acetate pathway is the one generating the more NADH in glycerol metabolism. Cluster 2 was more related to the OTUs that were over‐represented during classic fermentation and was composed of OTUs 6, 31 and 33. These OTUs were all estimated to have a lactate orientated metabolism. Cluster 3 grouped OTUs 3, 8 and 30 that were mostly under‐represented during EFG1. However, the metabolic profiles proposed by the model were all different between these three OTUs. Finally, cluster 4 gathered OTUs 5, 12, 34, 55 and 61 that were over‐represented during EF and estimated to have an ethanol or propionate metabolism. These OTUs (except for OTU 34) contained the worst PDO producers of the model, yet they represented altogether < 7.6% of the bacterial population in EF. Thus, they had a limited impact on EF metabolic pattern which was mainly explained by contributions of OTUs 1, 2 and 3 (see Table 1).

**Figure 3:**
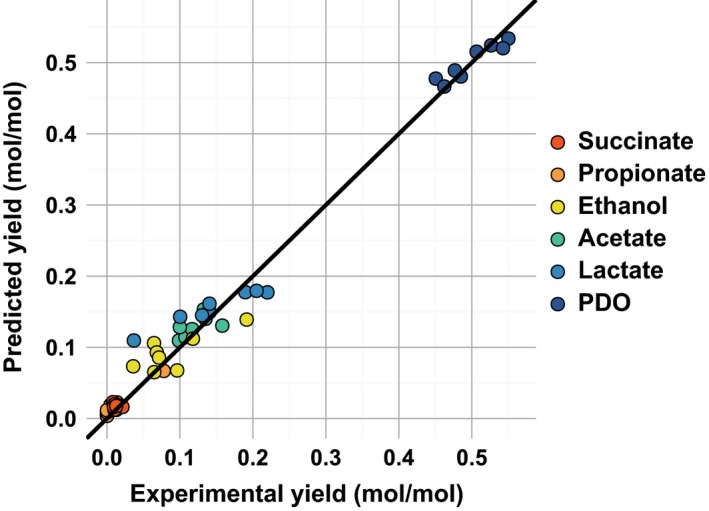
Global production yields predicted by the model in function of experimental production yields. Experimental yields correspond to the yields measured for each metabolite in each reactor whereas predicted yields are obtained by summing the contributions of each OTU as predicted by the model (six metabolites in eight reactors).

From the individually estimated metabolic pattern of each OTU, global yields could be assessed by the model and were compared with the actual yields (Fig. 3). The model was able to predict the global production yields satisfactorily and with high accuracy (root square mean errors, RMSE = 0.02 mol mol^−1^). Moreover, model accuracy and robustness were also validated by cross‐validation as reported in Appendix S2. Standard deviations provided in Fig. 2B show the yield distribution obtained during cross‐validation. It is noteworthy that the most dominant OTUs (i.e. OTUs 1, 2, 3, 6 and 34) had their metabolic profile nearly unchanged during cross‐validation.

### Population selection on *G. sulfurreducens* pre‐colonized electrodes

To go further in the analysis of the cathodic electro‐active biofilms, MiSeq sequencing was performed on cathode biofilm samples at the end of both EFG1 and EFG2. The bacterial population distribution within these biofilms is displayed in Appendix S3. After the first batch series (EFG1), *G. sulfurreducens* was still the dominant species on the cathode (59% of the total abundancy) while representing only 0.2% of the populations in the bulk phase. The other dominant species attached to the cathode (> 5% total abundancy) where OTUs 9, 14, 18 and 19 and were all affiliated to *Firmicutes*. OTU9 had 100% 16S rRNA sequence homology with *Clostridium celerecrescens* and represented 6.5% of the biofilm but was also present in significant amount in the bulk (5.0%). In contrast, OTUs 14, 18 and 19 (7.8, 6.5 and 5.0% of the total biofilm bacterial community respectively) were rare species in the bulk and accounted together for < 0.03% of the total bacterial community. OTU14 was an uncultured *Firmicutes* species and OTUs 18 and 19 were found to be related to *Geosporobacter subterraneus* and *Lutispora thermophila* (100 and 98% 16S rRNA sequence similarity respectively). OTUs 1 and 2, which were two of the dominant OTUs in the bulk, were also attached to the cathode but only accounted for, respectively, 2.0 and 4.1% of the biofilm meaning that the attachment on the working electrode was specific during EFG1. At the end of the second batch series (EFG2), *G. sulfurreducens* was not the dominant species anymore (12.9% of the biofilm community) and was less abundant than OTUs 1 and 9 (25.6 and 27.9% of the biofilm community respectively). OTUs 14, 18 and 19 were still accounting for a similar percentage of the biofilm population (9.1, 3.5 and 4.0% of the total biofilm bacterial community respectively).

## Discussion

### Selection effect versus individual metabolic shift

In mixed‐culture fermentations, changing an environmental parameter can induce at least two effects: (i) a metabolic shift in a part or in all the bacteria composing the mixed culture, resulting in a global change of the metabolism but not impacting the composition of the microbial community and/or (ii) a favoured or disfavoured selection of specific populations, resulting in a change of the microbial community and a subsequent change in global metabolism. Direct effects of an electrode on glycerol metabolism have already been shown in pure culture experiments using *Clostridium pasteurianum*, even with small currents (η_EF_ < 0.01) (Choi *et al*., 2014). Such a behaviour could be possible for some species in our experiments. However, the significant changes in the composition of the bacterial community between the different experiments and the robustness of the model tend to support a selection as a dominant effect rather than a shift in individual metabolic behaviour. Such strong changes in the composition of the bacterial community in electro‐fermentation of glycerol were also reported by Xafenias et al. (Xafenias *et al*., 2015). However, a highly negative potential was applied at the cathode in their experiments, resulting in the formation of significant amounts of H_2_ on the electrode surface. As hydrogen is a common electron shuttle supporting interspecies electron transfers (IET) (Kouzuma *et al*., 2015), it is not surprising that its continuous production at the cathode impacted the population composition. In the current study, electron inputs through the cathode were relatively low and the variations of microbial community composition in the bulk between fermentation controls (F) and electro‐fermentation reactors (EF) were not so sharp. By comparison, a significant change of the microbial community occurred when *G. sulfurreducens* pre‐colonized electrodes were used in electro‐fermentation (EFG1). Some OTUs estimated as being good PDO producers by the model were specifically enriched in the bulk during EFG1. Some of these OTUs, such as OTU2 (99% 16S rRNA sequence similarity with *Citrobacter freundii*), are related to species that are already known as efficient PDO producers (Drozdzynska *et al*., 2014; Lee *et al*., 2015). Others, such as OTU1, which belongs to the *Enterococcus* genus, became the most dominant OTU during EFG1 and counted for more than half of the microbial community. Species from the *Enterococcus* genus were never reported to produce PDO, even though *E. avium* along with *Enterococcus faecalis*,* Enterococcus faecium* and *Enterococcus gallinarum* were described as glycerol consumers (Collins *et al*., 1984). This genus is closely related to the *Lactobacillaceae* family in which some species, such as *Lactobacillus reuteri,* have already been characterized as PDO producers (Szymanowska‐Powałowska *et al*., 2013; Jolly *et al*., 2014). It is then possible that these two families share the same ability to synthetize PDO from glycerol and further investigations on species belonging to the *Enterococcus* genus could reveal new species of industrial interest.

### Electro‐fermentation redirects metabolic pathways

In the literature, the first electro‐fermentation results showed significant changes in mixed‐culture glycerol fermentation after adding electrodes in the bulk phase (Dennis *et al*., 2013; Zhou *et al*., 2013, 2015; Xafenias *et al*., 2015). The common strategy of these studies was to force an electron flow into the bioreactor by setting very low potentials at the WE (between −1.68 and −1.14 V versus SCE). This strategy led in some cases to an increase in PDO production yield (Zhou *et al*., 2013, 2015), a higher final concentration of PDO (Xafenias *et al*., 2015) along with changes in microbial population structures (Dennis *et al*., 2013; Xafenias *et al*., 2015). The conversion of glycerol (E_eq_ = 14) into PDO (E_eq_ = 16) requires only two electrons, meaning that a direct bio‐electrochemical conversion of glycerol into PDO would theoretically lead to a η_EF_ of 0.13 (two electrons over the 16 electrons contained in PDO). Electro‐fermentation efficiencies (η_EF_) estimated from these studies ranged between 0.34 and 0.79, indicating that the large majority of the cathodic electrons were not used for PDO production but were probably consumed for electrochemical splitting of water into H_2_, making these processes highly electron consuming.

In the present study, glycerol electro‐fermentation was conducted using a potential that could not support abiotic H_2_ production (see Fig. S7). Nonetheless, a metabolic shift was observed but was not related to more efficient PDO production. The productions of lactate, ethanol and propionate were indeed affected even at very low cathodic current (always more positive than −0.35 A m^−2^). As this low current density was likely due to a lack of electron mediators between the cathode and the bulk phase (e.g. H_2_, formate, metals, etc.), an alternative strategy had to be implemented in order to increase the bioavailability of cathodic electrons. Deutzmann and Spormann (2017) successfully used a Fe(0)‐corroding strain to catalyse electron transfer from the cathode to other microorganisms (e.g. homoacetogens, methanogens) through H_2_‐mediated electron transfers. *G. sulfurreducens* has also been described as being able to uptake electrons from a cathode (Choi and Sang, 2016) and to transfer electron to other species through IET (Cord‐Ruwisch *et al*., 1998; Shrestha and Rotaru, 2014; Lee *et al*., 2016; Rotaru and Shrestha, 2016). A recent study has even shown that *G. sulfurreducens* was able to transfer electrons to *C. pasteurianum* while the latter was fermenting glycerol (Moscoviz *et al*., 2017). Then, this species was a good candidate to facilitate electron transfers between the cathode and fermenters in the bulk.

In the current study, *G. sulfurreducens* had a significant impact on population selection in the bulk but failed to improve cathodic electron densities as less cathodic electrons were transferred in EFG1 than in EF. Thus, part of the population shift was likely due to specific interactions between some species from the bulk and *G. sulfurreducens* or other bacteria attached to the cathodes. This would also mean that the respective effect of a poised cathode presence and *G. sulfurreducens* pre‐colonization could be independent, or at least complementary. One hypothesis could be that some metabolites (e.g. acetate) were oxidized by *G. sulfurreducens* or other species on the biocathode, supporting their growth without consuming cathodic current. Part of the electrons released from this oxidation could be transferred to other species and would not be visible through electrochemical data but could cause population selection. Such interaction was demonstrated between *G. sulfurreducens* and *C. pasteurianum* (i.e. a fermentative species) in a co‐culture experiment during glycerol fermentation (Moscoviz *et al*., 2017) and could have occurred in the present study. Another species found on the biocathode, OTU9 (100% 16S rRNA sequence homology with *C. celerecrescens*), also displayed interesting behaviour as it dominated the biocathode in EFG1 and was also present in a significant amount in the bulk. Either such OTU was able to consume glycerol and interact with the biocathode, thus being an electro‐active fermenter, or it was growing by interacting with glycerol‐fermenting bacteria (e.g. by‐product consumption and interspecies electron transfer). However, the precise nature of interactions existing between the cathode, the cathodic biofilm and species of the bulk is not yet elucidated and should be the focus of further experiment (e.g. with simplified microbial communities).

### Perspectives

Electro‐fermentation is a promising concept that could help in specific mixed‐culture enrichments to improve metabolite production yields. The presence of an electrode inside a fermentation medium provides the opportunity to influence intracellular redox regulations without any addition of chemicals, but also to impact the microbial population structure during mixed‐culture processes. However, the main challenge regarding the future implementation of this concept is to find efficient catalysts able to ensure specific interactions between the electrochemical system and fermentative bacteria. Achieving high current densities is not relevant if this current is mainly used to sustain side‐reactions such as water electrolysis. The present study suggests that high current densities are not necessary to influence metabolic patterns and/or microbial population structure during glycerol mixed‐culture electro‐fermentation. Moreover, the addition of *G. sulfurreducens* as mediator had a significant impact on population selection and seemed to trigger the emergence of good PDO producers. Whether this is due to a better utilization of the cathodic current (i.e. transferred to fermenters) or to specific interactions independent from the cathode remains unclear but could open new opportunities regarding the use of electro‐active bacteria for a better control of fermentation processes.

## Methods

### Inoculum

The microbial inoculum used in this work corresponded to a mixed culture, originating from a long‐term continuous dark fermentation laboratory‐scale reactor fed with glycerol and operated at pH 6.5 under microaerophilic conditions, as described elsewhere (Moscoviz et al., 2016b). It was stored at 4°C for 2 months before use.

*G. sulfurreducens* DSM 12127 was purchased from the DSMZ (Braunschweig, Germany) collection and grown in 100 ml serum bottles sealed with butyl rubber stoppers and aluminium crimp caps, containing 50 ml of *Geobacter* medium (DSMZ Medium 826) prior to reactor inoculation.

### Fermentation medium

Unsterile minimal medium with no vitamins or yeast extract was used for all experiments. The composition of the fermentation medium (per litre of water) was as follows: 17.5 g glycerol, 1.75 g NH_4_Cl and 0.88 g NaCl (≥ 99%; Sigma‐Aldrich, Saint‐Louis, Missouri, USA). 10 ml of a trace element solution (1.5 g l^−1^ Nitrilotriacetic acid; 3.0 g l^−1^ MgSO_4_.7H_2_O; 0.50 g l^−1^ MnSO_4_.H_2_O; 1.0 g l^−1^ NaCl; 0.10 g l^−1^ FeSO_4_.7H_2_O; 0.18 g l^−1^ CoSO_4_.7H_2_O; 0.10 g l^−1^ CaCl_2_.2H_2_O; 0.18 g l^−1^ ZnSO_4_.7H_2_O; 0.01 g l^−1^ CuSO_4_.5H_2_O; 0.02 g l^−1^ KAl(SO_4_)_2_.12H_2_O; 0.01 g l^−1^ H_3_BO_3_; 0.01 g l^−1^ Na_2_MoO_4_.2H_2_O; 0.03 g l^−1^ NiCl_2_.6H_2_O; 0.30 mg l^−1^ Na_2_SeO_3_.5H_2_O; 0.40 mg l^−1^ Na_2_WO_4_.2H_2_O) and 100 mM phosphate buffer were added.

### Electro‐fermentation set‐up

Batch fermentation and electro‐fermentation experiments were performed in duplicates in potentiostat‐controlled H‐type reactors. All conditions were carried out sequentially in the following order: F, EF, EFG1 and EFG2 with 10 days between inoculation of F and EFG1. The dual chambers contained 900 ml of working volume with 100 ml of headspace in each half‐cell. The chambers were separated with a cation exchange membrane (Fumasep^®^ FAA‐3‐PK‐130, Fuma‐tech GmbH, St. Ingbert, Germany). The temperature was maintained at 37°C and pH was automatically regulated at 7.0 with NaOH 2M (pH probe InPro 4260i, Mettler Toledo). Initial biomass corresponded to 140 ml of the microbial inoculum centrifuged at 12 000 *g* for 15 min. The pellet was then suspended in the culture medium to reach a volatile solid content of 0.06 ± 0.00%_total mass_. Anaerobic conditions were established just after inoculation by flushing the media with high purity N_2_ (> 99.995%) for at least 30 min. Working electrode (WE) and counter electrodes were composed of 2.5 cm*2.5 cm*0.25 cm planar graphite (Goodfellow Cambridge Ltd., Huntingdon, UK) and 3 cm*2 cm 90% platinum – 10% iridium grids (Goodfellow Cambridge Ltd.) respectively. When a potential was applied, the WE was set at a fixed applied potential of −900 mV versus SCE using a VMP3 potentiostat/galvanostat (BioLogic Science Instruments, France). For fermentation experiments (F), electrodes were present but the electrical circuit was left open. In EFG1, WE were first colonized with *G. sulfurreducens* (See Appendix S1). A second electro‐fermentation batch series (EFG2) was also carried out using the bulk of EFG1 as inoculum (10% w/w). The two working electrodes at the end of EFG1 were used as pre‐colonized electrodes during EFG2. All the other parameters were the same as the first batch series.

### Analytical methods

Concentrations of glycerol, PDO and organic acids were measured by HPLC equipped with a refractive index detector (Waters R410), as described elsewhere (Moscoviz et al., 2016b). Samples were first centrifuged at 12 000 *g* for 15 min, and the supernatants were filtered with 0.2 μm syringe filters prior to analysis. HPLC analysis was performed at a flow rate of 0.4 ml min^−1^ on an Aminex HPX‐87H, 300 × 7.8 mm (Bio‐Rad, Hercules, California, USA) column at a temperature of 35°C. H_2_SO_4_ at 4 mM was used as the mobile phase.

### Microbial community analysis

In order to analyse microbial community structures, liquid samples from the bulk and biofilm samples from the WE were taken in the different experimental conditions. Liquid samples consisted of 2 ml collected in the bulk of each reactor (replicates of F, EF, EFG1 and EFG2) right after inoculation (initial biomass) and at the end of the experiments (final biomass). Biofilm samples were only taken from WEs of EFG1 and EFG2 at the end of the experiments. A sterile scalpel blade was used to scratch about 25 mm² of each WE. Then, the biomass obtained was suspended in 2 ml of sterile water. Both liquid and biofilm samples were centrifuged at 12 000 *g* for 15 min and pellets were stored at −20°C.

DNA was extracted and amplified as described elsewhere (Moscoviz et al., 2016b). The community composition was evaluated using MiSeq v3 (Illumina, San Diego, California, USA) with 2 × 300 bp paired‐end reads at the GenoToul platform ( http://www.genotoul.fr). Final sequences sets were retrieved after demultiplexing, cleaning, clustering (97%) and affiliating them using Mothur (Schloss *et al*., 2009). Sequences have been submitted to GenBank under the accession numbers No. KX031993–KX032512.

### Model assumptions

A linear inverse model consisting in a linear equation system constrained by an inequation system was used to estimate the metabolic pattern of each OTU found during sequencing. Only the OTUs with abundancy higher than 1% in at least one reactor were selected (15 OTUs). The model assumptions were as follows: (i) each OTU has a constant metabolic behaviour, (ii) each OTU contributes to the total metabolite production at a ratio equal to its abundancy, as the initial microbial biomass concentration can be neglected when compared to microbial growth (i.e. an OTU representing 30% of a reactor community will produce 30% of the metabolites of the reactor), (iii) each OTU has a balanced metabolism, that is production and consumption of NADH and ATP are equal and (iv) each OTU has closed electron and carbon mass balances. All these assumptions were converted into one linear equation system and one inequation system that are detailed in the Appendix S2.

In summary, a linear system of 108 equations and 165 inequations was obtained to estimate the 135 unknown parameters. This under‐determined linear problem was solved using the function ‘lsei’ of the package ‘limSolve’ version 1.5.5.1 (Soetaert *et al*., 2009) on the R 3.2.3 software (R Development Core Team 2010). The function used pseudo‐inverse matrices to solve the linear system and provided a unique solution that corresponded to the least square solution.

### Model cross‐validation

The model was validated using a k‐fold cross‐validation. The model had 48 equations issued from the experimental observations, with only eight independent reactors (six equations per reactor). The cross‐validation was performed by removing all the six equations of one reactor and calibrating the model on the 42 other equations. A root mean squared error of cross‐validation (RMSE_CV_) was then calculated by comparing the predicted global production yields of the removed reactor with its experimental values. A standard deviation for each Y_ij_ was also extracted from the cross‐validation to assess the sensitivity of the model to one particular reactor. Results of the cross‐validation are reported in Appendix S2.

### Statistical analysis

To help analysing the results obtained from the linear model, a clustering of the OTUs was performed based on their normalized bulk abundances. A value of *k* = 4 provided clusters that could be easily interpreted. The hierarchical clustering was made with the function ‘kmeans’ of the package ‘stats’ with the R 3.1.3 software with 100 iterations.

The Pearson correlations and significance calculations were made with the R 3.1.3 software (R Development Core Team 2010). For correlation coefficient calculations, the function ‘rcorr’ of the package Hmisc was used. Significance levels were assessed using 9999 random permutations with the function ‘sample’ of the package combinat.

## Conflict of interests

None declared.

## Supporting information


**Appendix S1.**
**Fig. S1.** Current production during colonization of working electrodes by pure cultures of *Geobacter sulfurreducens*.
**Fig. S2.** Mean glycerol consumption and metabolite production during open‐circuit experiments (F).
**Fig. S3.** Mean glycerol consumption and metabolite production during electro‐fermentation experiments at a working potential of −900 mV versus SCE (EF)
**Fig. S4.** Mean glycerol consumption and metabolite production during the first batch series of electro‐fermentation experiments at a working potential of −900 mV versus SCE and a *G. sulfurreducens* pre‐colonized electrode (EFG1)
**Fig. S5.** Mean glycerol consumption and metabolite production during the second batch series of electro‐fermentation experiments at a working potential of −900 mV versus SCE and a *G. sulfurreducens* pre‐colonized electrode (EFG2)
**Fig. S6.** Representative chronoamperometry curves of electro‐fermentation experiments.
**Fig. S7** Abiotic CV control using the same reactor configuration and medium as during electro‐fermentation experiments. The vertical black line corresponds to the potential chosen for electro‐fermentation experiments.
**Fig. S8.** Correlations between PDO yield and abundances of OTU1 and OTU13 (*P*‐values of 0.032 and 0.005 respectively).
**Appendix S2.**
**Fig. S9.** Theoretical range of production yields for the different metabolites considered in the linear model.
**Table S1.** Electron mass balances calculated from the metabolites measured after substrate depletion.
**Table S2.** Inverse model errors of prediction.
**Table S3.** Yields predicted by the model for each OTU sorted by decreasing PDO yield.

**Appendix S3.** Interactive Krona pie chart representing the mean of the bacterial population distribution of the inoculum and obtained in the different experimental conditions after substrate depletion: Fermentation (F), Electro‐fermentation (EF), EF+Geobacter‐Serie1 (EFG1) and EF+Geobacter‐Serie2 (EFG2).
