# Supplementary material for: Electro‐fermentation triggering population selection in mixed‐culture glycerol fermentation
Source: Microb Biotechnol. 2017 Jul 11;11(1):74–83. doi: 10.1111/1751-7915.12747 (PMC5743810; doi:10.1111/1751-7915.12747)
Supplement: Supplementary file 1 — Appendix S1. Fig. S1. Current production during colonization of working electrodes by pure cultures of Geobacter sulfurreducens. Fig. S2. Mean glycerol consumption and metabolite production during open‐circuit experiments (F). Fig. S3. Mean glycerol consumption and metabolite production during electro‐fermentation experiments at a working potential of −900 mV versus SCE (EF) Fig. S4. Mean glycerol consumption and metabolite production during the first batch series of electro‐fermentation experiments at a working potential of −900 mV versus SCE and a G. sulfurreducens pre‐colonized electrode (EFG1) Fig. S5. Mean glycerol consumption and metabolite production during the second batch series of electro‐fermentation experiments at a working potential of −900 mV versus SCE and a G. sulfurreducens pre‐colonized electrode (EFG2) Fig. S6. Representative chronoamperometry curves of electro‐fermentation experiments. Fig. S7 Abiotic CV control using the same reactor configuration and medium as during electro‐fermentation experiments. The vertical black line corresponds to the potential chosen for electro‐fermentation experiments. Fig. S8. Correlations between PDO yield and abundances of OTU1 and OTU13 (P‐values of 0.032 and 0.005 respectively). Appendix S2. Fig. S9. Theoretical range of production yields for the different metabolites considered in the linear model. Table S1. Electron mass balances calculated from the metabolites measured after substrate depletion. Table S2. Inverse model errors of prediction. Table S3. Yields predicted by the model for each OTU sorted by decreasing PDO yield. [file MBT2-11-74-s001.docx]

**Appendix S1.
Fig. S1.** Current production during colonization of working electrodes by pure cultures of *Geobacter sulfurreducens*.
**Fig. S2.** Mean glycerol consumption and metabolite production during open-circuit experiments (F).
**Fig. S3.** Mean glycerol consumption and metabolite production during electro-fermentation experiments at a working potential of −900 mV versus SCE (EF)
**Fig. S4.** Mean glycerol consumption and metabolite production during the first batch series of electro-fermentation experiments at a working potential of −900 mV versus SCE and a *G. sulfurreducens* pre-colonized electrode (EFG1)
**Fig. S5.** Mean glycerol consumption and metabolite production during the second batch series of electro-fermentation experiments at a working potential of −900 mV versus SCE and a *G. sulfurreducens* pre-colonized electrode (EFG2)
**Fig. S6.** Representative chronoamperometry curves of electro-fermentation experiments.
**Fig. S7** Abiotic CV control using the same reactor configuration and medium as during electro-fermentation experiments. The vertical black line corresponds to the potential chosen for electro-fermentation experiments.
**Fig. S8.** Correlations between PDO yield and abundances of OTU1 and OTU13 (*P*-values of 0.032 and 0.005 respectively).
**Appendix S2.
Fig. S9.** Theoretical range of production yields for the different metabolites considered in the linear model.
**Table S1.** Electron mass balances calculated from the metabolites measured after substrate depletion.
**Table S2.** Inverse model errors of prediction.
**Table S3.** Yields predicted by the model for each OTU sorted by decreasing PDO yield.
