# Supplementary material for: Electro‐fermentation triggering population selection in mixed‐culture glycerol fermentation
Source: Microb Biotechnol. 2017 Jul 11;11(1):74–83. doi: 10.1111/1751-7915.12747 (PMC5743810; doi:10.1111/1751-7915.12747)
Supplement: Supplementary file 2 — Appendix S3. Interactive Krona pie chart representing the mean of the bacterial population distribution of the inoculum and obtained in the different experimental conditions after substrate depletion: Fermentation (F), Electro‐fermentation (EF), EF+Geobacter‐Serie1 (EFG1) and EF+Geobacter‐Serie2 (EFG2). [file MBT2-11-74-s002.html]

Javascript must be enabled to view this page.

xml version='1.0' encoding='ASCII'?


count

Inoculum
Fermentation-Final
Electro-fermentation-Final
EF+Geobacter-Serie1-Final
EF+Geobacter-Serie1-Cathode
EF+Geobacter-Serie2-Final
EF+Geobacter-Serie2-Cathode


1
1
1
1
1
1
1


0.726108648
0.31913249
0.420702907
0.594518605
0.348230896
0.579475488
0.777176085


0.140824069
0.202117099
0.227826752
0.480515442
0.020632872
0.548679311
0.260211014


0.140733599
0.202117099
0.227826752
0.480362141
0.020623971
0.548625034
0.260211014


0.134528588
0.20145537
0.226813892
0.478013425
0.01992078
0.547346319
0.256953881


0.134488383
0.20145537
0.226813892
0.478013425
0.019742757
0.547307005
0.256234514


0.0000402048
0
0
0
0.000178023
0.0000393134
0.000719367


0.006029541
0.000418692
0.00101286
0.001476056
0.000302639
0.0000975559
0.000179842


0.006029541
0.000418692
0.00101286
0.001460876
0.000258133
0.0000838047
0.000159859


0
0
0
0.0000151805
0.0000445
0.0000137512
0.00002


0.0000823094
0.000212561
0
0.000849888
0.000400552
0.001171574
0.003077292


0.0000823094
0.000212561
0
0.000849888
0.000400552
0.001171574
0.003077292


0.0000931599
0
0
0
0
0
0


0.0000931599
0
0
0
0
0
0


0
0
0
0.0000227707
0
0
0


0
0
0
0.0000227707
0
0
0


0
0.000030476
0
0
0
0
0


0
0.000030476
0
0
0
0
0


0
0
0
0
0
0.00000958583
0


0
0
0
0
0
0.00000958583
0


0.0000810456
0
0
0.000153301
0.0000089
0.0000542772
0


0
0
0
0.0000834927
0
0.00000958583
0


0
0
0
0.0000834927
0
0.00000958583
0


0.0000502656
0
0
0
0.0000089
0
0


0.0000502656
0
0
0
0.0000089
0
0


0
0
0
0
0
0.000017189
0


0
0
0
0
0
0.000017189
0


0.00003078
0
0
0.0000698083
0
0.0000275024
0


0.00002394
0
0
0.0000345164
0
0.0000275024
0


0
0
0
0.000030361
0
0
0


0.00000683999
0
0
0.00000493092
0
0
0


0.0000094248
0
0
0
0
0
0


0.0000094248
0
0
0
0
0
0


0.0000094248
0
0
0
0
0
0


0.477408288
0.100876207
0.10375253
0.093883764
0.213520851
0.027142214
0.392334745


0.477408288
0.100876207
0.10375253
0.093883764
0.213138101
0.027142214
0.391995044


0.32069139
0.010926753
0.042616502
0.014033736
0.000356046
0.002699831
0.00231796


0.313349446
0.010589974
0.041603223
0.013295375
0.00031154
0.002658292
0.002258013


0.001452412
0.000144536
0.000253477
0.000434418
0
0.000035148
0.00002


0.0000215509
0.000030476
0
0.0000371758
0
0
0


0.000193254
0.0000101587
0.0000337132
0.0000398351
0
0
0


0.003350925
0.000122161
0.000270124
0.000222001
0.0000445
0.00000639055
0.00004


0.002224265
0.0000192886
0.000455965
0
0
0
0


0.0000441816
0.0000101587
0
0
0
0
0


0.000025968
0
0
0.00000493092
0
0
0


0
0
0
0
0
0
0


0.000029388
0
0
0
0
0
0


0.071898591
0.047971345
0.040534693
0.067407815
0.065770617
0.022479824
0.290004796


0.0084727
0.037880304
0.022886191
0.050924364
0.064889403
0.019035409
0.288106466


0.054635444
0.009881181
0.016973716
0.015567577
0.000703191
0.003422048
0.001638558


0.008744794
0.00020986
0.000674787
0.000915874
0.000178023
0.0000223669
0.000259771


0.0000031416
0
0
0
0
0
0


0.0000356712
0
0
0
0
0
0


0.00000683999
0
0
0
0
0
0


0.0000822384
0
0
0.000202277
0.064916107
0.0000415386
0.034929262


0.0000822384
0
0
0.000202277
0.064916107
0.0000415386
0.034929262


0.000125664
0
0
0.000228816
0.049597223
0.0000744614
0.039804972


0.000125664
0
0
0.000228816
0.049597223
0.0000744614
0.039804972


0.045677849
0.007012318
0.006959144
0.004886854
0.003284525
0.000531258
0.008312685


0.005530163
0.00146465
0.001063639
0.000643232
0.0000089
0.000132946
0.0000599


0.025390303
0.003937324
0.004054161
0.002687895
0.00015132
0.000227507
0.0000999


0.002245908
0.000192629
0.00038812
0.000160891
0
0.000029
0


0.007753448
0.000856415
0.000777391
0.000936095
0.002955183
0.000080852
0.007893054


0.004307389
0.000460741
0.000658872
0.000446219
0.000169122
0.0000609528
0.000219807


0.0000527712
0.0000101587
0.0000169612
0.0000125212
0
0
0


0.000331939
0
0
0
0
0
0.00004


0.0000493849
0.0000192886
0
0
0
0
0


0
0.000030476
0
0
0
0
0


0.0000165432
0
0
0
0
0
0


0
0.0000406347
0
0
0
0
0


0.024770634
0.031819043
0.010178952
0.003674353
0.000471761
0.00017323
0.000359683


0.021802303
0.031676178
0.009976254
0.003540776
0.000436156
0.00017323
0.000339701


0.002955764
0.000142864
0.000202698
0.000133577
0.0000356
0
0.00002


0.0000125664
0
0
0
0
0
0


0.008989491
0.00228146
0.002668991
0.002697036
0.0000623
0.00098603
0.000119894


0.008690349
0.002162642
0.002668991
0.002553597
0.0000534
0.000886292
0.000119894


0.000299142
0.000118818
0
0.000143439
0.0000089
0.0000997386
0


0.001635131
0.000243037
0.000253372
0.000327489
0.016876586
0.0000543197
0.007813124


0.000100531
0
0
0.000148758
0.011313365
0.000035148
0.003896571


0.001518056
0.000223748
0.000253372
0.000128259
0.003133206
0
0.002337943


0.0000062832
0
0
0
0.002430015
0.0000127811
0.001478699


0.00001026
0.0000192886
0
0.0000504724
0
0.00000639055
0.0000999


0.0000498253
0
0
0.0000629936
0.007272242
0.0000447339
0.004116378


0.0000121261
0
0
0.0000531317
0.007218835
0.0000319528
0.004116378


0.0000376992
0
0
0.00000986184
0.0000534
0.0000127811
0


0.002416342
0.000451611
0.000101663
0.000274357
0.0000089
0.0000299701
0.0000599


0.002409502
0.000357483
0.000101663
0.000274357
0.0000089
0.0000299701
0


0
0
0
0
0
0
0


0.00000683999
0.0000941285
0
0
0
0
0.0000599


0.000354797
0
0.000371787
0.0000151805
0.003346833
0.0000100709
0.002737591


0.000354797
0
0.000371787
0.0000151805
0.003346833
0.0000100709
0.002737591


0.000467529
0.0000192886
0
0.0000504724
0
0
0


0
0.00000642955
0
0.0000455415
0
0
0


0.000408196
0
0
0.00000493092
0
0
0


0.0000593328
0.0000128591
0
0
0
0
0


0.0000251328
0
0
0
0.001174952
0.0000137512
0.001298857


0.0000251328
0
0
0
0.001174952
0.0000137512
0.001298857


0.000188837
0.000151351
0.0000674263
0.000022383
0
0.00000319528
0


0.000188837
0.000151351
0.0000674263
0.000022383
0
0.00000319528
0


0.00000342
0
0
0
0
0
0


0.00000342
0
0
0
0
0
0


0.000031416
0
0
0
0
0
0.000119894


0.000031416
0
0
0
0
0
0.000119894


0
0
0
0
0.00038275
0
0.000339701


0
0
0
0
0.00038275
0
0.000339701


0
0
0
0
0.00038275
0
0.000339701


0.000327005
0
0
0.001430292
0.112848814
0.000220474
0.122672049


0.000327005
0
0
0.001430292
0.112848814
0.000220474
0.122672049


0.000327005
0
0
0.001430292
0.112848814
0.000220474
0.122672049


0.000327005
0
0
0.001430292
0.112848814
0.000220474
0.122672049


0.101220177
0.01562675
0.013412686
0.017788304
0.001077039
0.003397128
0.001198945


0.101220177
0.01562675
0.013412686
0.017788304
0.001077039
0.003397128
0.001198945


0.074776753
0.009192705
0.008405662
0.010675675
0.000471761
0.002375108
0.000459596


0.036475334
0.00480377
0.004433482
0.005795635
0.000160221
0.001298397
0.000179842


0.023981814
0.001857881
0.002010224
0.002261122
0.000222529
0.000307147
0.000179842


0.014285093
0.002520896
0.001843646
0.002604124
0.000089
0.000769564
0.0000999


0.0000242521
0.0000101587
0.0000677403
0
0
0
0


0
0
0.0000505698
0.0000147928
0
0
0


0.00001026
0
0
0
0
0
0


0.026416342
0.006434045
0.005007024
0.007097449
0.000605278
0.001022021
0.000739349


0.019132823
0.004831545
0.003637141
0.005803504
0.000391651
0.000792531
0.000479578


0.005798099
0.001414628
0.00113274
0.000894043
0.00015132
0.000153816
0.000219807


0.001051742
0.000138107
0.000203221
0.000322115
0.0000623
0.0000528221
0.00004


0.000433678
0.0000497647
0.0000339225
0.0000777863
0
0.000022852
0


0.0000270816
0
0
0.0000151805
0
0
0


0.0000270816
0
0
0.0000151805
0
0
0


0.000194017
0
0.074866854
0
0
0.000023337
0.000239789


0.000194017
0
0.074866854
0
0
0.000023337
0.000239789


0.000194017
0
0.074866854
0
0
0.000023337
0.000239789


0.000194017
0
0.074866854
0
0
0.000023337
0.000239789


0.006135092
0.000512435
0.000844085
0.000900803
0.00015132
0.0000130236
0.000519543


0.006135092
0.000512435
0.000844085
0.000900803
0.00015132
0.0000130236
0.000519543


0.006135092
0.000512435
0.000844085
0.000900803
0.00015132
0.0000130236
0.000519543


0.005620915
0.000329964
0.000692166
0.00080972
0.00015132
0.0000130236
0.000519543


0.000514177
0.00018247
0.000151919
0.000091083
0
0
0


0.071614964
0.115812936
0.191630552
0.251992089
0.636583738
0.096846358
0.160139078


0.049110376
0.100327379
0.174438242
0.230343271
0.044594775
0.088638694
0.029494045


0.020208488
0.08060861
0.152778719
0.22126859
0.044345543
0.070798142
0.025937175


0.020208488
0.08060861
0.152778719
0.22126859
0.044345543
0.070798142
0.025937175


0.02008769
0.08060861
0.152745006
0.192082249
0.041568383
0.066219844
0.025437615


0.000120798
0
0.0000337132
0.02917116
0.00277716
0.004578298
0.00049956


0
0
0
0.0000151805
0
0
0


0.003759434
0.018292567
0.012994638
0.008302137
0.0000712
0.017528186
0.00347694


0.003759434
0.017988836
0.012994638
0.008302137
0.0000712
0.017528186
0.00347694


0.003759434
0.017988836
0.012994638
0.008302137
0.0000712
0.01748592
0.003456958


0
0
0
0
0
0
0.00002


0
0
0
0
0
0.0000422662
0


0
0.000284443
0
0
0
0
0


0
0.000253967
0
0
0
0
0


0
0.000030476
0
0
0
0
0


0
0.0000192886
0
0
0
0
0


0
0.0000192886
0
0
0
0
0


0.022835313
0.000831983
0.008376962
0.000646945
0.000178023
0.000269858
0.0000599


0.02264766
0.000700306
0.008376962
0.000646945
0.000178023
0.000260272
0.0000599


0.016887374
0.00041702
0.000473449
0.000601404
0.000178023
0.0000366032
0.0000599


0.005760286
0.000283286
0.007903513
0.0000455415
0
0.000223669
0


0.000187653
0.000131677
0
0
0
0.00000958583
0


0.000187653
0.000070725
0
0
0
0.00000958583
0


0
0.0000609521
0
0
0
0
0


0.002294575
0.00035041
0.000287922
0.000125599
0
0.0000425087
0.00002


0.002025991
0.000238665
0.000254
0.000125599
0
0.0000425087
0.00002


0.002025991
0.000238665
0.000254
0.000125599
0
0.0000425087
0.00002


0.000268584
0.000111745
0.0000339225
0
0
0
0


0.000268584
0.000111745
0.0000339225
0
0
0
0


0.0000125664
0.000243808
0
0
0
0
0


0.0000125664
0.000243808
0
0
0
0
0


0.0000125664
0.000243808
0
0
0
0
0


0.000792623
0.000030476
0
0.001632181
0.591125551
0.000274024
0.129645912


0.000731993
0
0
0.001632181
0.591125551
0.000274024
0.129645912


0.000731993
0
0
0.001632181
0.591027638
0.000274024
0.129486052


0.000731993
0
0
0.001632181
0.591027638
0.000274024
0.129486052


0
0
0
0
0.0000979
0
0.000159859


0
0
0
0
0.0000979
0
0.000159859


0.0000606303
0
0
0
0
0
0


0.0000606303
0
0
0
0
0
0


0.0000606303
0
0
0
0
0
0


0
0.000030476
0
0
0
0
0


0
0.000030476
0
0
0
0
0


0
0.000030476
0
0
0
0
0


0.020439735
0.00928529
0.005830067
0.019511525
0.000836708
0.007425391
0.000939174


0.014066182
0.005354653
0.004289107
0.012621328
0.000836708
0.005167151
0.000819279


0.012442678
0.003527762
0.001606189
0.007865227
0.000258133
0.001252238
0.000179842


0.005379083
0.002002931
0.000491457
0.006545574
0.0000534
0.000672451
0.000159859


0.007063595
0.001373479
0.001114732
0.0012745
0
0.000573397
0.00002


0
0.000110717
0
0.0000151805
0.000204727
0
0


0
0
0
0.0000299732
0
0.00000639055
0


0
0.0000406347
0
0
0
0
0


0.001620362
0.001826891
0.002665956
0.004733718
0.000578575
0.003901162
0.000639437


0
0
0
0.00000759025
0
0
0


0.001344832
0.001826891
0.002632139
0.004695766
0.000578575
0.003901162
0.000639437


0.00025843
0
0.0000338178
0.000030361
0
0
0


0.0000171
0
0
0
0
0
0


0.0000031416
0
0.0000169612
0.000022383
0
0.0000137512
0


0.0000031416
0
0.0000169612
0.00000759025
0
0
0


0
0
0
0.0000147928
0
0.0000137512
0


0.005158656
0.00321953
0.00149039
0.006700883
0
0.002235873
0.0000799


0.005158656
0.00321953
0.00149039
0.006700883
0
0.002235873
0.0000799


0.00049782
0.000469743
0.000797178
0.006061528
0
0.002128246
0


0.00464485
0.000768073
0.0006595
0.000582068
0
0.000087
0


0.0000065616
0.000929969
0
0.0000421067
0
0.0000206268
0.0000599


0
0.000467299
0
0
0
0
0


0.0000094248
0.000335236
0.0000168566
0
0
0
0.00002


0
0.0000507934
0
0
0
0
0


0
0.0000561942
0.0000168566
0.0000151805
0
0
0


0
0.0000711107
0
0
0
0
0


0
0.0000406347
0
0
0
0
0


0
0.000030476
0
0
0
0
0


0.001214898
0.0000203174
0.0000505698
0.000189314
0
0.0000223669
0


0.001214898
0.0000203174
0.0000505698
0.000189314
0
0.0000223669
0


0.001214898
0.0000203174
0.0000505698
0.000189314
0
0.0000127811
0


0
0
0
0
0
0.00000958583
0


0
0.00023365
0
0
0
0
0.00004


0
0.000223491
0
0
0
0
0


0
0.000223491
0
0
0
0
0


0
0.0000101587
0
0
0
0
0.00004


0
0.0000101587
0
0
0
0
0.00004


0
0.000294602
0
0
0
0
0


0
0.000294602
0
0
0
0
0


0
0.000294602
0
0
0
0
0


0
0.000162539
0
0
0
0
0


0
0.000162539
0
0
0
0
0


0
0.000162539
0
0
0
0
0


0.001272229
0.006078363
0.011362243
0.000505112
0.0000267
0.000508249
0.0000599


0.0000706119
0.000173598
0
0.000447825
0
0.0000319528
0


0.0000065616
0.0000964432
0
0.000440234
0
0.0000255622
0


0.0000065616
0.0000900136
0
0.000425054
0
0.0000255622
0


0
0.00000642955
0
0.0000151805
0
0
0


0.0000606303
0.0000192886
0
0.00000759025
0
0.00000639055
0


0
0
0
0.00000759025
0
0.00000639055
0


0.0000606303
0.0000192886
0
0
0
0
0


0.00000342
0.0000257182
0
0
0
0
0


0.00000342
0.0000257182
0
0
0
0
0


0
0.0000321477
0
0
0
0
0


0
0.0000321477
0
0
0
0
0


0.001006035
0.005553455
0.011024797
0.00000986184
0
0.000415386
0.0000599


0.001006035
0.005553455
0.011024797
0.00000986184
0
0.000415386
0.0000599


0.000976368
0.005522979
0.011024797
0.00000986184
0
0.000415386
0.0000599


0.0000296664
0
0
0
0
0
0


0
0.000030476
0
0
0
0
0


0
0.0000192886
0
0
0
0
0


0
0.0000192886
0
0
0
0
0


0
0
0
0
0
0
0


0
0.0000192886
0
0
0
0
0


0.0000062832
0.000205745
0
0.0000246546
0
0
0


0.0000062832
0.000205745
0
0.0000246546
0
0
0


0.0000062832
0.000205745
0
0.0000246546
0
0
0


0.000175898
0.0000663529
0.000337446
0
0.0000267
0.0000471591
0


0.000175898
0.0000663529
0.000337446
0
0.0000267
0.0000368457
0


0.000145363
0.0000101587
0.000337446
0
0
0.00000687559
0


0.0000062832
0
0
0
0
0
0


0.0000242521
0.0000257182
0
0
0
0.0000299701
0


0
0.000030476
0
0
0
0
0


0
0
0
0
0.0000267
0
0


0
0
0
0
0
0.0000103134
0


0
0
0
0
0
0.0000103134
0


0.0000134016
0.0000406347
0
0.0000227707
0
0
0


0.0000134016
0.0000406347
0
0.0000227707
0
0
0


0.0000134016
0
0
0
0
0
0


0
0.0000406347
0
0
0
0
0


0
0
0
0.0000227707
0
0
0


0
0.0000192886
0
0
0
0.0000137512
0


0
0.0000192886
0
0
0
0.0000137512
0


0
0.0000192886
0
0
0
0.0000137512
0


0
0.0000914281
0
0
0
0
0


0
0.0000914281
0
0
0
0
0


0
0.0000914281
0
0
0
0
0


0
0.0000914281
0
0
0
0
0


0.199501216
0.548368748
0.38416488
0.14654127
0.015078553
0.320116374
0.061585804


0.199076093
0.546288147
0.384130958
0.146427804
0.015034047
0.320116374
0.061585804


0.199076093
0.546288147
0.384130958
0.146427804
0.015034047
0.320116374
0.061585804


0.05359528
0.407401104
0.292913567
0.006681207
0.00038275
0.280391172
0.040484374


0.020047587
0.40521403
0.290698448
0.002941201
0.000089
0.279832982
0.039804972


0.025817448
0.001519944
0.001589332
0.002555814
0.000169122
0.000239076
0.000399648


0.006320785
0.000446725
0.000524647
0.000878753
0.000089
0.000298973
0.000139877


0.001290437
0.0000524651
0.00010114
0.000272806
0.0000356
0.00000319528
0.00004


0
0.0000203174
0
0
0
0.00000319528
0.00002


0.0000342
0.000147622
0
0.0000326326
0
0.0000137512
0.0000799


0.0000848232
0
0
0
0
0
0


0.11716575
0.136524957
0.089338775
0.13787302
0.014526681
0.039430636
0.020901607


0.11716575
0.136524957
0.089338775
0.13787302
0.014526681
0.039430636
0.020901607


0.016751297
0.001732119
0.001404852
0.001223252
0.0000534
0.000138324
0


0.016751297
0.001732119
0.001404852
0.001223252
0.0000534
0.000138324
0


0.01141627
0.000617108
0.000473763
0.00055963
0.0000712
0.000122833
0


0.011413128
0.000617108
0.000473763
0.00055963
0.0000712
0.000122833
0


0.0000031416
0
0
0
0
0
0


0
0
0
0.0000151805
0
0
0.00002


0
0
0
0.0000151805
0
0
0.00002


0.0000462096
0.0000128591
0
0.0000755147
0
0.0000302126
0.000179842


0
0
0
0.00000759025
0
0
0


0.0000462096
0.0000128591
0
0.0000679245
0
0.0000302126
0.000179842


0.000101287
0
0
0
0
0
0


0.000101287
0
0
0
0
0
0


0
0
0
0
0
0.00000319528
0


0
0
0
0
0
0.00000319528
0


0.000174367
0.001271121
0
0.0000759025
0
0
0


0.000174367
0.001271121
0
0.0000759025
0
0
0


0.000164107
0
0
0.0000531317
0
0
0


0.000164107
0
0
0.0000531317
0
0
0


0
0.000325078
0
0.0000227707
0
0
0


0
0.000213332
0
0
0
0
0


0
0.0000812694
0
0
0
0
0


0
0
0
0.0000227707
0
0
0


0
0.000030476
0
0
0
0
0


0
0.00019533
0
0
0
0
0


0
0.00019533
0
0
0
0
0


0.00001026
0.000507934
0
0
0
0
0


0.00001026
0.000507934
0
0
0
0
0


0
0.000100558
0
0
0
0
0


0
0.000100558
0
0
0
0
0


0
0.0000711107
0
0
0
0
0


0
0.0000711107
0
0
0
0
0


0
0.0000406347
0
0
0
0
0


0
0.0000406347
0
0
0
0
0


0
0.000030476
0
0
0
0
0


0
0.000030476
0
0
0
0
0


0.00023791
0.000227477
0.0000339225
0.0000326326
0.0000445
0
0


0.00023791
0.000227477
0.0000339225
0.0000326326
0.0000445
0
0


0.00023791
0.000146208
0.0000339225
0.0000326326
0.0000445
0
0


0.0000593328
0.000030476
0
0.0000250423
0
0
0


0.000105693
0
0
0.00000759025
0
0
0


0.0000634598
0.000115732
0.0000339225
0
0.0000445
0
0


0.0000094248
0
0
0
0
0
0


0
0.0000507934
0
0
0
0
0


0
0.0000507934
0
0
0
0
0


0
0.000030476
0
0
0
0
0


0
0.000030476
0
0
0
0
0


0.0000128448
0.000115732
0
0.00000493092
0
0
0


0.0000128448
0.000115732
0
0.00000493092
0
0
0


0.0000128448
0.000115732
0
0.00000493092
0
0
0


0.00000342
0.000115732
0
0.00000493092
0
0
0


0.0000094248
0
0
0
0
0
0


0
0.000354525
0
0
0
0
0


0
0.000354525
0
0
0
0
0


0
0.000354525
0
0
0
0
0


0
0.000354525
0
0
0
0
0


0
0.000111745
0
0
0
0
0


0
0.000111745
0
0
0
0
0


0
0.000111745
0
0
0
0
0


0
0.000111745
0
0
0
0
0


0.000219361
0.013850011
0.001998599
0.005607496
0.0000979
0.002950397
0.000679402


0.000219361
0.013850011
0.001998599
0.005607496
0.0000979
0.002950397
0.000679402


0.000219361
0.013850011
0.001998599
0.005599906
0.0000979
0.002950397
0.000679402


0.000219361
0.013850011
0.001998599
0.005599906
0.0000979
0.002950397
0.000679402


0.000219361
0.013850011
0.001998599
0.005599906
0.0000979
0.002950397
0.000679402


0
0
0
0.00000759025
0
0
0


0
0
0
0.00000759025
0
0
0


0
0
0
0.00000759025
0
0
0
