# Supplementary figures and images for: Electro‐fermentation triggering population selection in mixed‐culture glycerol fermentation
Source: Microb Biotechnol. 2017 Jul 11;11(1):74–83. doi: 10.1111/1751-7915.12747 (PMC5743810; doi:10.1111/1751-7915.12747)

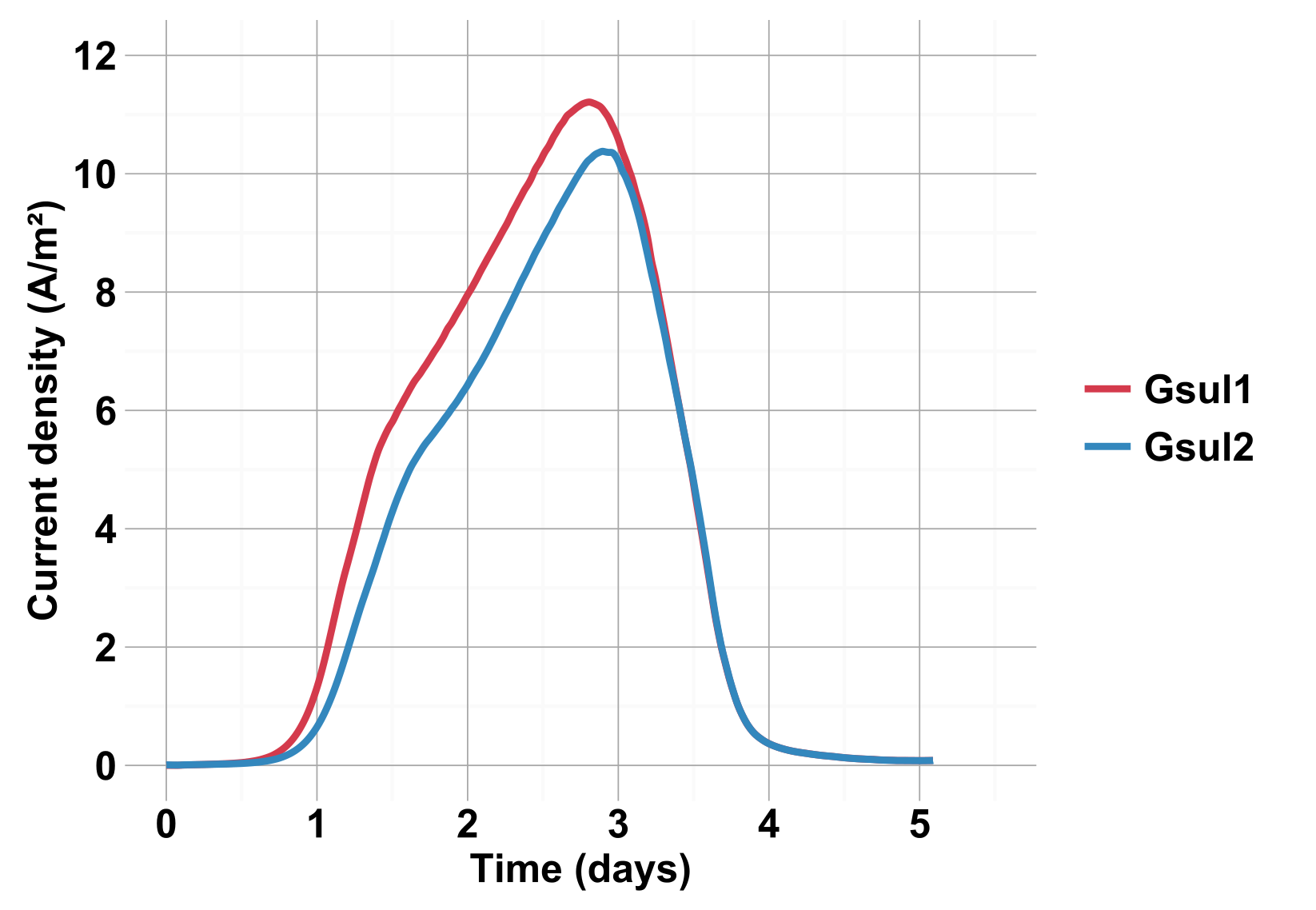

Supplement: Supplementary file 3 [file MBT2-11-74-s003.png]

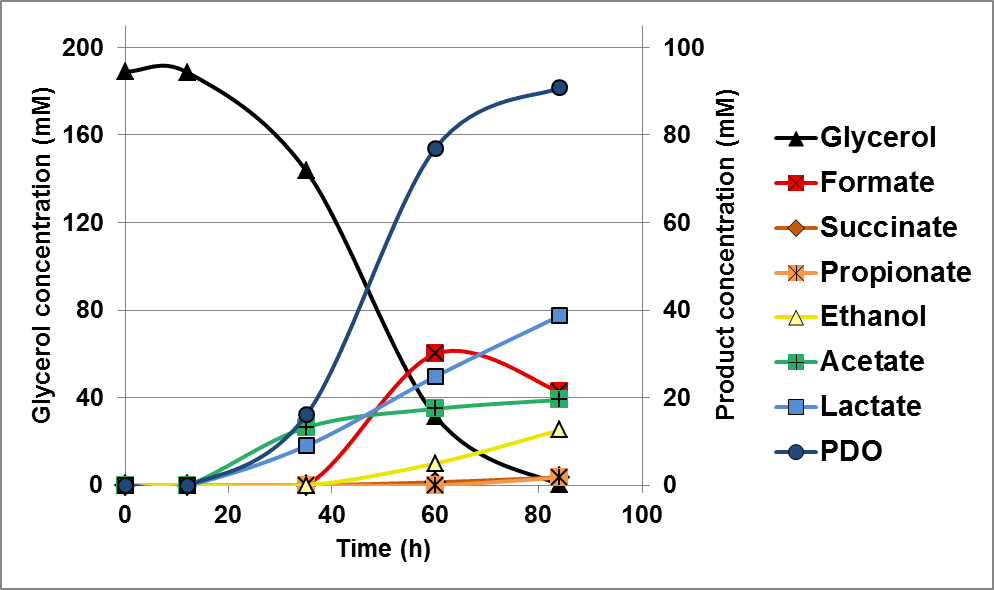

Supplement: Supplementary file 4 [file MBT2-11-74-s004.png]

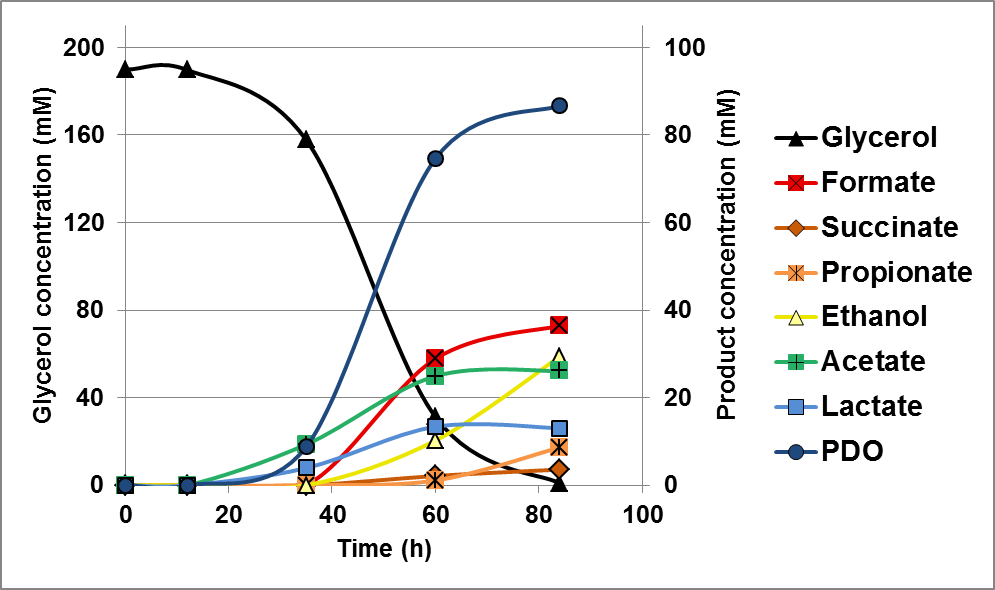

Supplement: Supplementary file 5 [file MBT2-11-74-s005.png]

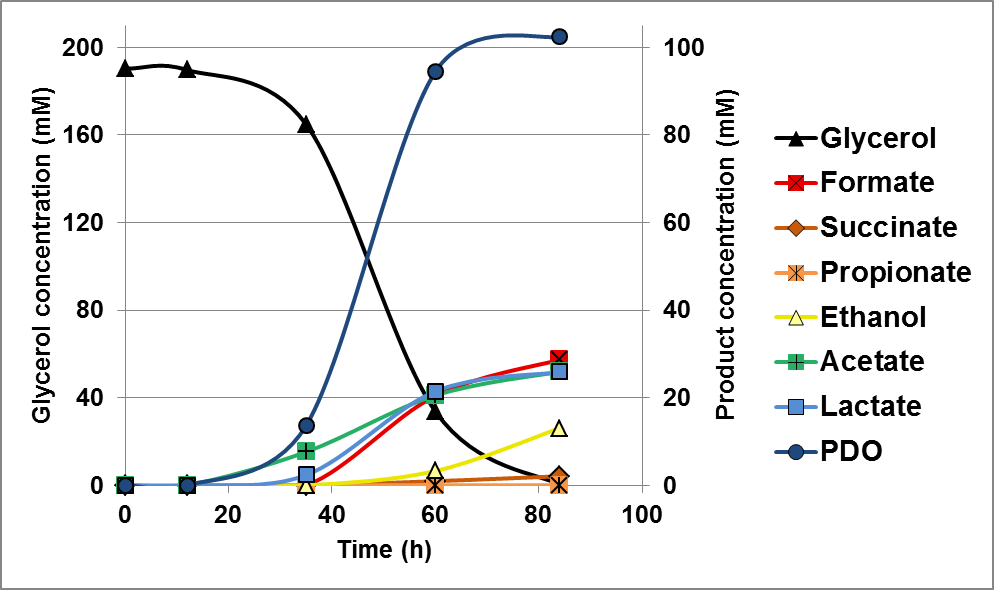

Supplement: Supplementary file 6 [file MBT2-11-74-s006.png]

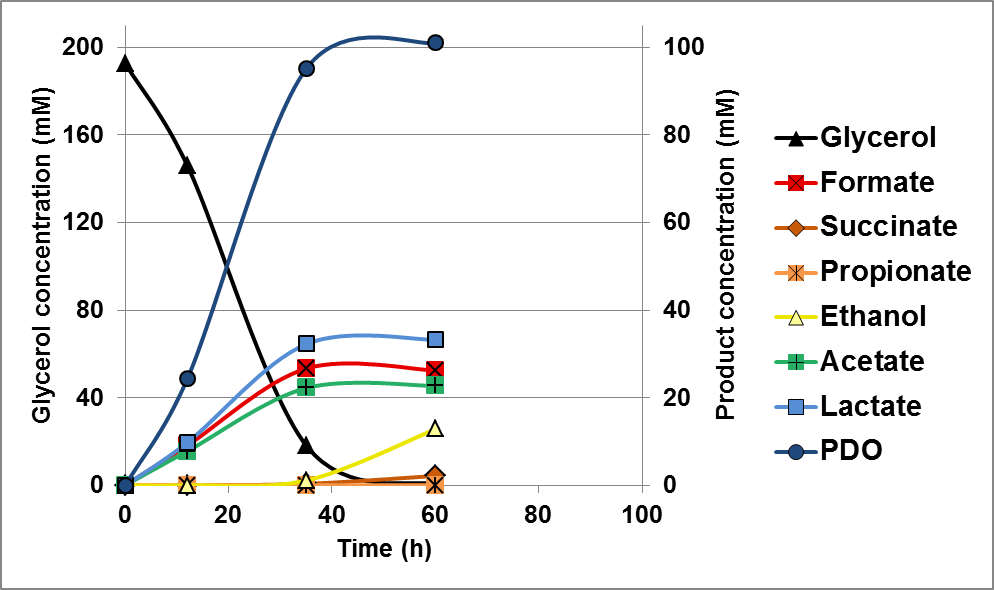

Supplement: Supplementary file 7 [file MBT2-11-74-s007.png]

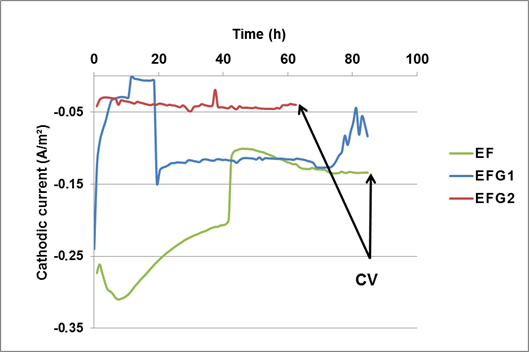

Supplement: Supplementary file 8 [file MBT2-11-74-s008.jpg]

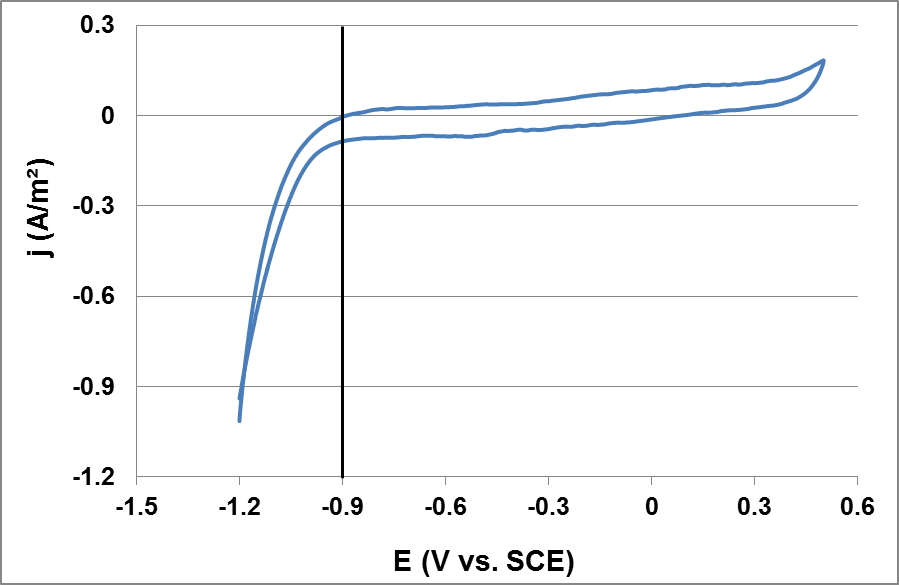

Supplement: Supplementary file 9 [file MBT2-11-74-s009.png]

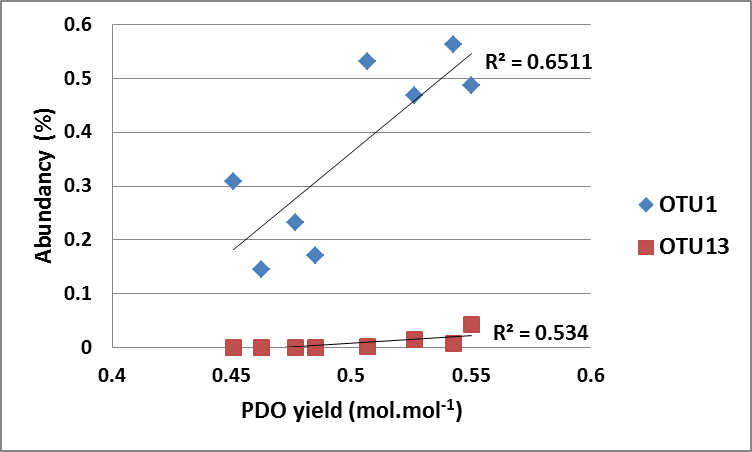

Supplement: Supplementary file 10 [file MBT2-11-74-s010.png]

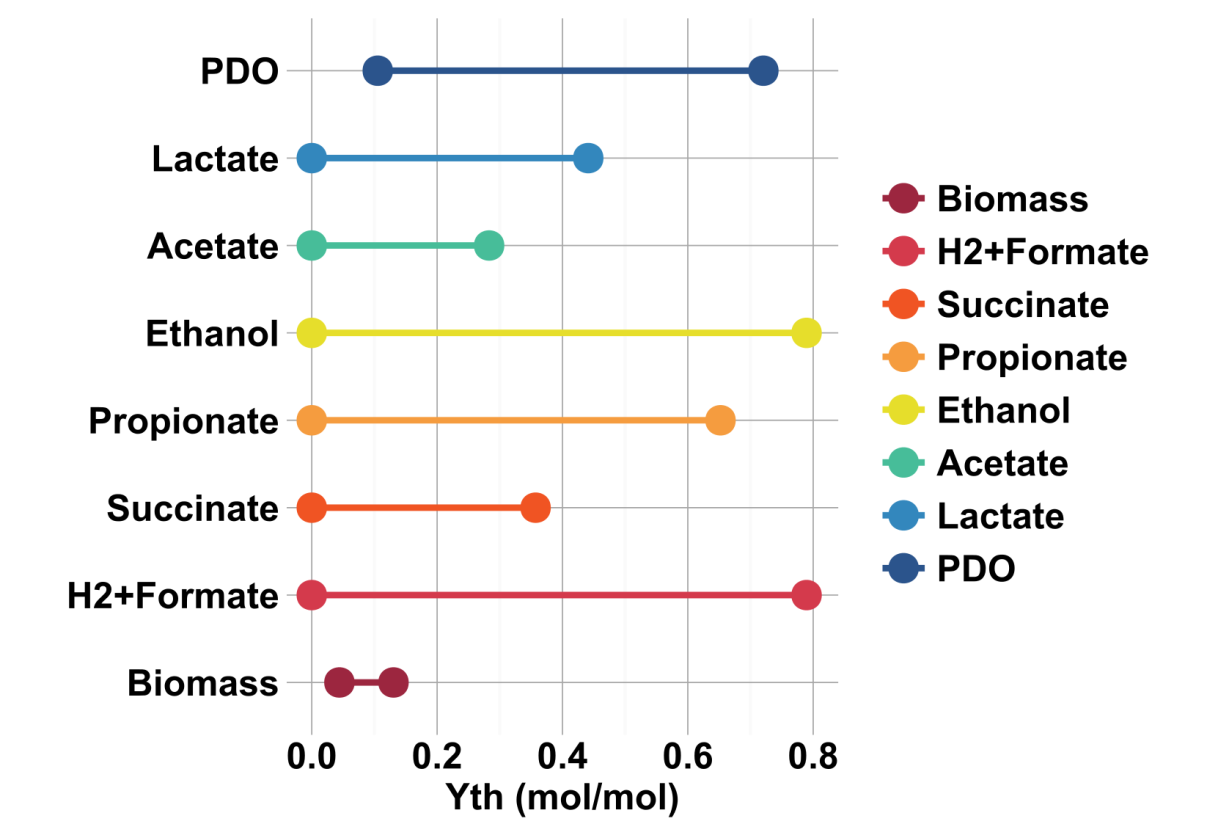

Supplement: Supplementary file 11 [file MBT2-11-74-s011.png]
