# Supplementary material for: Electro‐fermentation triggering population selection in mixed‐culture glycerol fermentation
Source: Microb Biotechnol. 2017 Jul 11;11(1):74–83. doi: 10.1111/1751-7915.12747 (PMC5743810; doi:10.1111/1751-7915.12747)
Supplement: Supplementary file 12 [file MBT2-11-74-s012.docx]

**Table S1** Electron balances calculated from the metabolites measured after glycerol depletion.

| Reactor | Succinate  (%) | Lactate  (%) | Formate  (%) | Acetate  (%) | PDO  (%) | Propionate  (%) | Ethanol  (%) | Butyrate  (%) | Biomass*  (%) | Recovery  (%) |
| --- | --- | --- | --- | --- | --- | --- | --- | --- | --- | --- |
| F-1 | 0.8 | 16.3 | 1.6 | 5.6 | 55.4 | 0.5 | 5.5 | 0 | 5.7 | 91.5 |
| F-2 | 1.0 | 18.8 | 1.6 | 6.1 | 54.5 | 1.4 | 5.9 | 0 | 6.6 | 95.9 |
| EF-1 | 1.7 | 8.6 | 3.5 | 6.7 | 51.5 | 1.3 | 16.4 | 0 | 6.9 | 96.6 |
| EF-2 | 2.1 | 3.2 | 2.0 | 9.0 | 52.8 | 7.8 | 10.1 | 1.3 | 8.2 | 96.5 |
| EFG1-1 | 1.1 | 12.1 | 2.1 | 7.6 | 62.9 | 0 | 5.6 | 0 | 5.9 | 97.2 |
| EFG1-2 | 1.0 | 11.2 | 2.2 | 8.0 | 60.2 | 0 | 6.1 | 0 | 6.0 | 94.7 |
| EFG2-1 | 1.0 | 12.0 | 1.8 | 7.7 | 62.0 | 0 | 3.1 | 0 | 5.5 | 93.2 |
| EFG2-2 | 1.3 | 17.6 | 2.1 | 5.7 | 57.9 | 0 | 8.2 | 0 | 6.3 | 99.0 |

Results are normalized on initial glycerol electron content. The biomass was estimated from the ATP production associated to the different metabolites production. F: Classic fermentation. EF: Electro-fermentation. EFG1-2: Successive batches of electro-fermentation with *G. sulfurreducens* pre-colonized cathode.
