# Supplementary material for: Electro‐fermentation triggering population selection in mixed‐culture glycerol fermentation
Source: Microb Biotechnol. 2017 Jul 11;11(1):74–83. doi: 10.1111/1751-7915.12747 (PMC5743810; doi:10.1111/1751-7915.12747)
Supplement: Supplementary file 13 [file MBT2-11-74-s013.docx]

**Table S2** Inverse model errors of prediction

|  | RMSE  (mol/mol) | RMSE_CV_  (mol/mol) |
| --- | --- | --- |
| Global | 0.021 | 0.042 |
| PDO | 0.014 | 0.032 |
| Lactate | 0.034 | 0.063 |
| Acetate | 0.016 | 0.033 |
| Ethanol | 0.029 | 0.063 |
| Propionate | 0.009 | 0.014 |
| Succinate | 0.007 | 0.012 |
