# Supplementary material for: Electro‐fermentation triggering population selection in mixed‐culture glycerol fermentation
Source: Microb Biotechnol. 2017 Jul 11;11(1):74–83. doi: 10.1111/1751-7915.12747 (PMC5743810; doi:10.1111/1751-7915.12747)
Supplement: Supplementary file 14 [file MBT2-11-74-s014.docx]

**Table S3** Yields predicted by the model for each OTU sorted by decreasing PDO yield

| OTU n° | PDO  (mol/mol) | Lactate  (mol/mol) | Acetate  (mol/mol) | Ethanol  (mol/mol) | Propionate  (mol/mol) | Succinate  (mol/mol) | H_2_+Formate  (mol/mol) | Biomass  (mol/mol) |
| --- | --- | --- | --- | --- | --- | --- | --- | --- |
| 13 | 0.64 | 0.00 | 0.28 | 0.00 | 0.00 | 0.00 | 0.28 | 0.06 |
| 1 | 0.55 | 0.17 | 0.15 | 0.04 | 0.00 | 0.01 | 0.19 | 0.05 |
| 2 | 0.53 | 0.00 | 0.22 | 0.14 | 0.00 | 0.02 | 0.36 | 0.06 |
| 6 | 0.53 | 0.19 | 0.13 | 0.00 | 0.03 | 0.03 | 0.13 | 0.06 |
| 9 | 0.50 | 0.44 | 0.00 | 0.00 | 0.00 | 0.00 | 0.00 | 0.04 |
| 31 | 0.50 | 0.44 | 0.00 | 0.00 | 0.00 | 0.00 | 0.00 | 0.04 |
| 33 | 0.50 | 0.44 | 0.00 | 0.00 | 0.00 | 0.00 | 0.00 | 0.04 |
| 8 | 0.49 | 0.44 | 0.00 | 0.01 | 0.00 | 0.00 | 0.01 | 0.04 |
| 34 | 0.48 | 0.00 | 0.18 | 0.00 | 0.21 | 0.00 | 0.18 | 0.10 |
| 3 | 0.46 | 0.17 | 0.10 | 0.11 | 0.03 | 0.03 | 0.21 | 0.07 |
| 55 | 0.21 | 0.00 | 0.00 | 0.00 | 0.56 | 0.00 | 0.00 | 0.17 |
| 61 | 0.21 | 0.00 | 0.00 | 0.00 | 0.56 | 0.00 | 0.00 | 0.17 |
| 5 | 0.11 | 0.00 | 0.00 | 0.79 | 0.00 | 0.00 | 0.79 | 0.08 |
| 12 | 0.11 | 0.00 | 0.00 | 0.79 | 0.00 | 0.00 | 0.79 | 0.08 |
| 30 | 0.11 | 0.00 | 0.00 | 0.79 | 0.00 | 0.00 | 0.79 | 0.08 |
